# Snakebite-Associated Infections: A Systematic Review and Meta-Analysis

**DOI:** 10.4269/ajtmh.23-0278

**Published:** 2024-03-19

**Authors:** D. Katterine Bonilla-Aldana, Jorge Luis Bonilla-Aldana, Juan R. Ulloque-Badaracco, Ali Al-kassab-Córdova, Enrique A. Hernandez-Bustamante, Esteban A. Alarcon-Braga, Abdelmonem Siddiq, Vicente A. Benites-Zapata, Alfonso J. Rodriguez-Morales, Camila Luna, Jose A. Suarez

**Affiliations:** ^1^ Research Unit, Universidad Continental, Huancayo, Peru;; ^2^ Grupo de Investigación en Ciencias Animales Macagual, Universidad de La Amazonia, Florencia, Colombia;; ^3^ Escuela de Medicina, Universidad Peruana de Ciencias Aplicadas, Lima, Peru;; ^4^ Centro de Excelencia en Investigaciones Económicas y Sociales en Salud, Universidad San Ignacio de Loyola, Lima, Peru;; ^5^ Grupo Peruano de Investigación Epidemiológica, Unidad para la Generación y Síntesis de Evidencias en Salud, Universidad San Ignacio de Loyola, Lima, Perú;; ^6^ Sociedad Científica de Estudiantes de Medicina de la Universidad Nacional de Trujillo, Peru;; ^7^ Faculty of Pharmacy, Mansoura University, Mansoura, Egypt;; ^8^ Unidad de Investigación para la Generación y Síntesis de Evidencias en Salud, Vicerrectorado de Investigación, Universidad San Ignacio de Loyola, Lima, Peru;; ^9^ Grupo de Investigación Biomedicina, Faculty of Medicine, Fundación Universitaria Autónoma de las Américas-Institución Universitaria Visión de las Américas, Pereira, Colombia;; ^10^ Faculty of Health Sciences, Universidad Científica del Sur, Lima, Peru;; ^11^ Gilbert and Rose-Marie Chagoury School of Medicine, Lebanese American University, Beirut, Lebanon;; ^12^ Universidad de Panama, Investigator 1 of the SNI, Senacyt, Panama City, Panama

## Abstract

Snakebites still constitute a significant public health problem in developing countries and are considered a neglected tropical condition by the WHO. Snake accidents are associated with substantial morbidity and mortality and may produce secondary complications, such as severe infections. The objective of this systematic review was to determine the prevalence of snakebite infections and characterize the bacteria isolated from these infections. A systematic literature review in five databases was carried out to assess the prevalence of snakebite infection. A meta-analysis was performed using a random-effects model to calculate the pooled prevalence and 95% CIs. Cochran’s *Q* test and the *I*^2^ statistic were used to assess between-study heterogeneity. The pooled prevalence of infection due to snakebite was 27.0% (95% CI: 22.0–32.0%), with high heterogeneity among studies (*I*^2^ = 99.7%). The prevalence was higher in Asia (32%) than in the Americas (21%). Snakebite infections required surgical interventions in 68% (95% CI: 37.0–98.0%). The leading group of pathogens identified corresponded to Gram-negative bacteria (63%), particularly *Morganella morganii* (32%), but also, Gram-positive cocci (40%), especially *Enterococcus* spp. (23%) and *Staphylococcus aureus* (15%). However, multiple other pathogens, including anaerobes, were found. A high prevalence of snakebite-associated infection has been described, primarily due to *M. morganii*, with the corresponding implications for empirical therapy. Rational use of antimicrobials is recommended, and this should guide initial empirical treatment. Moreover, isolation and identification of the possible bacteria present in snakebite wounds is recommended in all cases to confirm or rule out associated infection.

## INTRODUCTION

Snakebite is the only neglected tropical disease of noninfectious origin included in the WHO list.^1^^–^^3^ Although the ecoepidemiology of snakebite is similar to zoonotic infectious diseases,^4^ snakebite envenomation occurs after the inoculation of toxins into tissues by grooved fangs that may be contaminated by bacteria.^5^ Consequently, snake accidents are associated with significant general morbidity and mortality, producing secondary complications such as severe systemic and local septic infection.^1^^,^^6^

Animal venoms are considered sterile sources of antimicrobial compounds with intense bactericidal activity that disrupts the membrane of multidrug-resistant bacteria.^7^^,^^8^ In the case of snakebites, the cause of death is often due to a toxic hemorrhagic effect or a neurotoxic effect with a secondary bacterial infection.^7^^,^^9^

Of the five families of snakes that comprise the species capable of poisoning humans, Elapidae and Viperidae are the most important from a medical point of view.^10^ The Elapidae family includes cobras, kraits, mambas, coral snakes, Austro-Oceanic snakes, and sea snakes. The family Viperidae includes Old World vipers, rattlesnakes, moccasins, lancehead vipers, mamushis, habus, and other Eurasian vipers. Families of less medically critical venomous snakes are Lamprophiidae (Atractaspidinae; African/Middle Eastern burrowing asps) and Colubridae (now divided; snakes with opisthoglyphous dentition).^10^

Snakebites with cytotoxic and proteolytic effects cause lesion development and severe tissue necrosis at the bite site. In addition, dead tissue can become infected by bacteria from the oral cavity of the snake.^11^ The oral microbiota of snakes comprises a wide range of aerobic and anaerobic microorganisms, especially Gram-negative bacilli in the feces of snake-digested prey.^12^

The proteolytic properties of snake venom cause extensive tissue destruction and devitalization, predisposing the wound to bacterial infection.^12^ Wound infection after snakebite occurs in 9% to 77% of bitten individuals.^12^ The principal microorganisms responsible are *Aeromonas hydrophila*, *Morganella morganii*, *Klebsiella pneumoniae*, *Bacillus* sp., and *Enterococcus* spp.^13^

It has been reported that the oral cavity of the Russell’s viper harbors a diverse array of pathogenic bacteria, including Gram-negative genera (*Proteus* sp., *Pseudomonas* sp., *Salmonella* sp., *Providencia* sp., *Alcaligenes* sp., *Morganella* sp., and *Escherichia coli*) and Gram-positive genera (*Bacillus* and *Enterococcus* sp., *Staphylococcus* sp. and *Lysinobacillus* sp.).^14^ Another study identified a wide range of pathogenic bacteria, including *Salmonella arizonae*, *Pseudomonas stutzeri*, *Proteus penneri*, *Alcaligenes faecalis*, *Citrobacter diversus* and *Citrobacter freundii*, *Enterococcus faecalis*, *Bacillus anthracis*, *Staphylococcus sciuri*, and *Achromobacter xylosoxidans* were isolated as new additions to the floral diversity of the scale viper.^15^

Other authors analyzed tusk, tusk sheath, and venom cultures from 15 healthy, newly captured *Bothrops jararaca*. The bacteria most frequently found were group D streptococci (12 snakes), *Enterobacter* sp. (six), *Providencia rettgeri* (six), *Providencia* sp. (four), *E. coli* (four), *M. morganii* (three) and *Clostridium* sp. (five). The bacteria identified were similar to those found in the abscesses of patients bitten by *Bothrops*. Because these snake mouth bacteria can be inoculated during a snakebite, bacterial multiplication and infection can occur under favorable conditions.^16^

The objective of the present systematic review was to determine the prevalence of snakebite infections and the bacteria isolated.

## MATERIALS AND METHODS

This systematic review followed the Preferred Reporting Items for Systematic Reviews and Meta-Analysis (PRISMA) guidelines. In addition, the protocol of this study was registered in the PROSPERO database (ID: CRD42023391691).

### Information sources and search strategy.

The PubMed, Scopus, Web of Science, SciELO, and Embase databases were searched, with no language or geographic location restrictions (Supplementary Table 1). The search strategy was developed using the Peer Review of Electronic Search Strategies (PRESS) Checklist.^16^ Initially, the strategy was built in PubMed and was later modified to be adapted to other databases. Literature was searched from inception of the databases to December 22, 2022.

### Study selection and data extraction.

The following inclusion criteria were considered: 1) studies that assessed the prevalence of infection in snakebites; 2) studies that assessed the prevalence of the different bacteria identified in snakebites; 3) studies that assessed the prevalence of surgical intervention (including wound incision, pus drainage, debridement, and fasciotomy for necrotizing fasciitis or compartment syndrome) secondary to snakebite infections; 4) cohort, case–control, and cross-sectional studies; and 5) carried out in people of any age. We excluded the following studies: systematic reviews, scoping reviews, narrative reviews, conference abstracts, and case reports.

The bibliographic search results were uploaded to Rayyan QCRI software. Two researchers (A. Al-kassab-Córdova and E. A. Hernandez-Bustamante) independently screened all titles and abstracts. The remaining studies were retrieved in full text and independently assessed by the same researchers (see excluded articles by full text in Supplemental Table 2). Discussion with a third party (V. A. Benites-Zapata) resolved any reviewer disagreement. Articles that met the selection criteria were included in the systematic review. For each article selection phase, the Cohen’s kappa coefficient (Cohen’s κ) was used to determine the degree of agreement between the authors who screened the articles.^17^

The information on the selected articles was collected in a data extraction table developed in Microsoft Excel by two researchers (E. A. Hernandez-Bustamante and A. Siddiq). Finally, the extracted information was compared, and consensus resolved disagreements. The following information was collected: title, country, age, study design, gender, number of people presenting snakebite infection, bacteria identified in snakebite infection, and number and type of surgical interventions secondary to snakebite infection.

### Quality assessment of studies and publication bias.

Four researchers (J. R. Ulloque-Badaracco, A. Al-kassab-Córdova, and E. A. Hernandez-Bustamante) independently evaluated the included studies using the Newcastle–Ottawa Scale (NOS) for cohorts/case controls and the adapted NOS for cross-sectional studies.^17^^,^^18^ In both cases, a study with seven or more stars was deemed to be of high methodological quality or low risk of bias. In contrast, studies with fewer than seven were considered to be of low methodological quality or high risk of bias.

The current literature does not recommend the evaluation of publication bias in the case of systematic reviews of prevalence studies because there are no tests that correctly fit the proportional data.^19^^,^^20^

## STATISTICAL ANALYSES

The quantitative analyses were performed with Stata 16.0 (Stata Corporation, College Station, TX). A combined analysis of the studies that evaluated the prevalence of snakebite infection with its corresponding 95% CI was carried out. The random effects model (Dersimonian and Laird) was used. The 95% CI was calculated using the Clopper–Pearson method. Heterogeneity between studies was assessed using the *I*^2^ statistic and Cochran’s *Q* test. In the case of the *I*^2^ statistic, values greater than 60% were considered high heterogeneity. On the other hand, a *P*-value <0.05 was considered a sign of heterogeneity in the Cochran’s *Q* test.

Following the same methodology, meta-analyses of the prevalence of bacteria identified in snakebites and the prevalence of surgical intervention secondary to snakebite infection were also performed. In addition, subgroup analyses were carried out according to continents and snake families. Finally, sensitivity analyses were performed, excluding studies with a high risk of bias.

## RESULTS

### Study selection.

**Figure 1.**
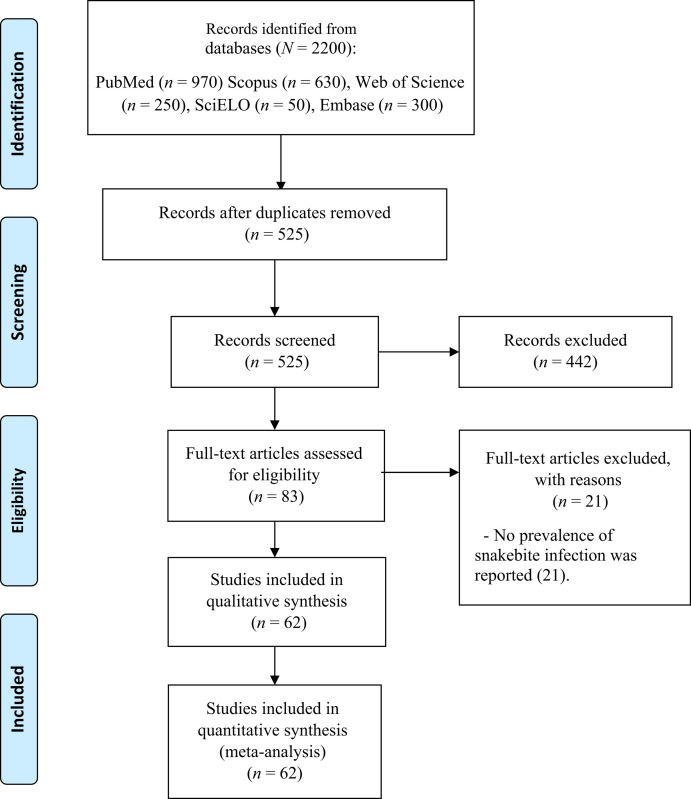
PRISMA flow diagram.

The literature search identified 2,200 records, of which 1,675 were removed due to duplication. After screening the studies by title/abstract (Cohen’s κ: 0.34), 83 articles remained. Finally, after full-text assessment (Cohen’s κ: 0.53), 62 studies were included in the meta-analysis and quantitative synthesis.^9^^,^^12^^,^^21^^–^^80^ References to the analyzed studies are usually not included. The selection process is summarized in Figure 1.

### Study characteristics.

The characteristics of the studies included are summarized in Table 1. Sixty-two studies (*N =* 84,296) were conducted between 1989 and 2022 in the following continents: Asia (33 studies), America (24 studies), Africa (four studies), and Europe (one study). All the studies defined the diagnosis of snakebite infection from a physical examination of the area affected by the bite with signs of an infected wound or progressive tissue necrosis. All the studies defined the diagnosis of snakebite infection as signs of an infected wound or progressive tissue necrosis on physical examination of the area affected by the bite.

**Table 1:** Characteristics of the studies included

Author	Year	Country	Participants (male/female)	Median/Mean/ Range Age (IQR/SD)	No. of Participants with SI	Prevalence of SI	No. of Participants Requiring Surgical Intervention for SI	Types of Bacteria in SI (*n/N*)	Bacteria Isolated in SI (*n/N*)
Chen et al.	2011	Taiwan	231 (144/87)	4–95	59	25.50%	26	-Gram-positive bacteria: 14/61 -Gram-negative bacteria: 39/61 -Anaerobic bacteria: 8/61	-*Enterococcus* species: 12/61 -*Morganella morganii*: 14/61 -*Proteus* species: 5/61 -*Pseudomonas aeruginosa*: 5/61 -*Shewanella* species: 3/61 -*Citrobacter* species: 4/61 -*Escherichia coli*: 2/61 -*Bacteroides fragilis*: 6/61 -*Klebsiella pneumoniae*: 1/61 -*Serratia* species: 2/61
Wagener et al.	2017	South Africa	164 (NR/NR)	NR	40	24.39%	NR	-Gram-positive bacteria: 13/48 -Gram-negative bacteria: 35/48 -Anaerobic bacteria: 0/48	-*Morganella morganii*: 20/48 -*Enterococcus* species: 15/48 -*Proteus* species: 12/48 -*Escherichia coli*: 2/48 -*Citrobacter* species: 2/48 -*Klebsiella pneumoniae*: 2/68
Huang et al.	2012	Taiwan	121 (74/47)	4–90	34	28.00%	24	-Gram-positive bacteria: 7/41 -Gram-negative bacteria: 33/41 -Anaerobic bacteria: 1/41	-*Morganella morganii*: 15/41 -*Aeromonas hydrophila*: 8/41 -*Enterococcus* species: 5/41 -*Proteus* species: 3/41 -*Escherichia coli*: 1/41 -*Shewanella* species: 1/41 -*Pseudomonas aeruginosa*: 1/41 -*Serratia* species: 2/41 -*Staphylococcus aureus*: 2/41
Blaylock et al.	1999	South Africa	310 (NR/NR)	NR	17	5.48%	14	-Gram-positive bacteria: 2/20 -Gram-negative bacteria: 18/20 -Anaerobic bacteria: 0/20	-*Morganella morganii*: 4/20 -*Proteus* species: 4/20 -*Escherichia coli*: 2/20 -*Serratia* species: 3/20 -*Citrobacter* species: 3/20
Ngo et al.	2020	Vietnam	46 (NR/NR)	NR	36	78.00%	NR	-Gram-positive bacteria: 26/46 -Gram-negative bacteria: 20/46	-Enterococcus species: 25/46 -*Morganella morganii*: 11/46 -*Proteus* species: 2/46 -*Klebsiella pneumoniae*: 1/46 -*Staphylococcus aureus*: 1/46
Hsieh et al.	2017	Taiwan	148 (100/48)	NR	42	28.00%	NR	-Gram-positive bacteria: 11/30 -Gram-negative bacteria: 16/30 -Anaerobic bacteria: 3/30	-*Morganella morganii*: 12/30 -*Aeromonas hydrophila*: 1/30 -*Bacteroides fragilis*: 3/30 -*Proteus* species: 3/30 -*Enterococcus* species: 11/30
Mao et al. (Cohort A)	2018	Taiwan	183 (116/67)	51.5 (18.5)	148	80.90%	21	-Gram-positive bacteria: 11/35 -Gram-negative bacteria: 24/35 -Anaerobic bacteria: 1/35	-*Morganella morganii*: 12/35 -*Aeromonas hydrophila*: 1/35 -*Enterococcus* species: 10/35 -*Proteus* species: 4/35 -*Escherichia coli*: 1/41 -*Shewanella* species: 1/35 -*Pseudomonas aeruginosa*: 1/35 -*Bacteroides fragilis*: 1/35 -*Serratia* species: 2/35
Mao et al. (Cohort B)	2016	Taiwan	112 (NR/NR)	NR	86	76.78%	NR	NR	
Houcke S et al.	2022	French Guiana	172 (119/53)	41 (28–52)	55	31.97%	43	-Gram-positive bacteria: 2/17 -Gram-negative bacteria: 15/17	-*Morganella morganii*: 12/17 -*Aeromonas hydrophila*: 1/17 -*Escherichia coli*: 1/17 -*Pseudomonas aeruginosa*: 1/17 -*Staphylococcus aureus*: 2/17
Lin et al.	2020	Taiwan	726 (506/220)	51.88 (17.42)	163	22.45%	NR	-Gram-positive bacteria: 11/20 -Gram-negative bacteria: 5/20 -Anaerobic bacteria: 4/20	-*Morganella morganii*: 3/20 -*Enterococcus* species: 3/20 -*Bacteroides fragilis*: 1/20 -*Aeromonas hydrophila*: 1/20 -*Staphylococcus aureus*: 2/20
Sasa et al.	2020	Costa Rica	475 (NR/NR)	NR	33	6.90%	NR	NR	NR
Weed et al.	1993	United States	72 (63/9)	NR	0	0%	NR	NR	NR
Clark et al.	1993	United States	41 (32/9)	NR	3	7.30%	NR	NR	NR
Kouyoumdjian et al.	1989	Brazil	22 (NR/NR)	NR	4	18.18%	NR	NR	NR
Magalhães et al.	2019	Brazil	70,816 (55,557/ 15,248)	NR	2639	3.72%	NR	NR	NR
Kriengkrairut et al.	2021	Thailand	123 (83/40)	52 (36–66)	8	6.50%	NR	NR	NR
Osmani et al.	2007	Pakistan	110 (72/38)	11-80	62	56.36%	NR	NR	NR
Nascimento et al.	2022	Brazil	3,297 (0/3,297)	28.3 (10.7)	178	5.90%	NR	NR	NR
Mendes et al.	2022	Brazil	127(101/26)	64 (50.4)	127	23.30%	NR	NR	NR
Sachett et al.	2017	Brazil	153/33	NR	74	39.80%	NR	-Gram-positive bacteria: 1/6 -Gram-negative bacteria: 5/6	-*Morganella morganii*: 5/6 -*Staphylococcus aureus*: 1/6
Ruha et al.	2017	United States	450 (312/138)	1-89	2	0.40%	NR	NR	NR
Hansdak et al.	1998	Nepal	52 (36/16)	13-64	10	19.00%	NR	NR	NR
Villanueva Forero et al.	2004	Peru	170 (107/63)	26.2 (17.95)	14	8.20%	NR	NR	NR
Otero et al. (Cohort A)	2002	Colombia	39 (31/8)	15–70	12	30.8%	NR	-Gram-positive bacteria: 9/10 -Gram-negative bacteria: 1/10	-*Morganella morganii*: 2/10 -*Proteus* species: 1/10 -*Aeromonas hydrophila*: 2/10 -*Staphylococcus aureus*: 2/10 -*Klebsiella pneumoniae*: 1/10
Otero et al. (Cohort B)	1992	Colombia	524 (NR/NR)	27 (NR)	56	10.60%	NR	NR	NR
Lopez et al.	2008	Colombia	48 (6/42)	24.6 (8–61)	16	33.30%	NR	-Gram-positive bacteria: 2/11 -Gram-negative bacteria: 9/11	-*Morganella morganii*: 5/11 -*Proteus* species: 1/11 -*Escherichia coli*: 1/11 -*Enterococcus* species: 1/11
Yeh et al.	2021	Taiwan	195 (144/51)	49.97 (17.42)	53	27.20%	NR	NR	NR
Frangides et al.	2005	Greece	147 (85/62)	48.11(17.71)	20	13.60%	NR	NR	NR
Silva et al.	2020	Brazil	144 (NR/NR)	NR	11	9.00%	NR	NR	NR
White et al.	2018	Myanmar	948 (580/368)	NR	82	8.80%	NR	NR	NR
Yakubu et al.	2018	Ghana	119 (83/36)	26.38 ± 16.46	42	35.30%	NR	NR	NR
Bhalla et al.	2014	India	150 (99/51)	NR	4	2.60%	NR	NR	NR
Looareesuwan et al.	1988	Thailand	46 (NR/NR)	1–81	6	13.00%	NR	NR	NR
Kumar et al. (Cohort A)	2019	India	58 (NR/NR)	NR	55	94.00%	NR	NR	NR
Tan et al.	2010	Singapore	52 (43/9)	13–69	2	3.84%	NR	NR	NR
Mohammed et al.	2022	Ethiopia	250 (202/48)	24 (22–26)	130	62.80%	NR	NR	NR
Murugan et al.	2015	India	82 (64/18)	14 − 65	48	58.54%	NR	NR	NR
Enzenhofer et al.	2018	Argentina	67 (51/47)	15-49	15	22.00%	NR	NR	NR
Ho et al.	2019	Taiwan	125 (88/38)	NR	0	0%	NR	NR	NR
Chew et al.	2011	Malaysia	260(154/106)	NR	13	5.00%	NR	NR	NR
Pradhan et al.	2022	India	88 (48/40)	NR	24	27.27%	NR	NR	NR
Kim K et al.	2020	Korea	61 (36/25)	61 (56 − 71)	6	9.80%	NR	NR	NR
Bhelkar et al.	2017	India	156 (100/56)	37.78 (14)	75	66.96%	NR	NR	NR
Lai et al.	2022	Taiwan	161 (114/47)	50.4 (17.7)	80	49.68%	72	-Gram-negative bacteria: 33/34 -Anaerobic bacteria: 1/34	-*Enterococcus* species: 12/34 -*Morganella morganii*: 11/34 -*Serratia* species: 2/34 -*Shewanella* species: 2/34 -*Aeromonas hydrophila*: 1/34 -*Citrobacter* species: 1/34 -*Proteus* species: 3/34 -*Bacteroides fragilis*: 1/34
Monteiro et al.	2012	India	31 (18/13)	19–65	29	93.50%	NR	NR	NR
Garg A et al.	2009	India	43 (31/12)	NR	NR	NR	NR	-Gram-positive bacteria: 17/53 -Gram-negative bacteria: 25/53	-*Staphylococcus aureus*: 17/53 -*Enterococcus* species: 4/53 -*Escherichia coli*: 8/53 -*Klebsiella pneumoniae*: 4/53 -*Proteus* species: 3/53 -*Morganella morganii*: 3/53 -*Pseudomonas aeruginosa*: 3/53
Lath et al.	2015	India	454 (312/142)	NR	138	30.39%	NR	NR	NR
Liu et al.	2012	Taiwan	10 (4/6)	42.5 (26–88)	NR	NR	NR	-Gram-negative bacteria: 24/24	-*Shewanella species*: 10/24 -*Morganella morganii*: 4/24 -*Enterococcus* species: 3/24 -*Bacteroides fragilis*: 2/24 -*Aeromonas hydrophila*: 1/24 -*Proteus* species: 1/24
Dookeram et al.	2022	Trinidad and Tobago	28 (22/6)	NR	1	3.4%	NR	NR	NR
Kumar et al. (Cohort B)	2021	India	300 (209/91)	NR	18	6.00%	NR	NR	NR
Chatterjee et al.	2022	India	94 (58/36)	7.4 (NR)	16	17.02%	NR	NR	NR
Ashok et al.	2021	India	91 (NR/NR)	NR	32	35.1%%	NR	NR	NR
Miah et al.	2009	Bangladesh	46 (36/10)	34.9 (16.2)	7	15.2%%	NR	NR	NR
Reddy et al.	2019	India	60(25/35)	NR	10	17.00%	NR	NR	NR
Chinga et al.	2004	Ecuador	41 (NR/NR)	NR	29	70.73%	NR	NR	NR
Kerrigan (Cohort A) et al.	1997	Ecuador	114 (63/51)	3–84	9	8.00%	NR	-Gram-positive bacteria: 4/11 -Gram-negative bacteria: 7/11	-*Escherichia coli*: 3/11 -*Staphylococcus aureus*: 4/11 -*Proteus* species: 1/11 -*Klebsiella pneumoniae*: 2/11
Saravu et al.	2012	India	76 (46/30)	16–82	8	10.52%	NR	NR	NR
Morejon-Garcia et al.	2006	Brazil	30 (23/7)	NR	3	10.00%	3	NR	NR
Matute-Martinez et al.	2016	Honduras	59 (NR/NR)	24 (NR)	6	10.16%	NR	NR	NR
García-Willis et al.	2009	Mexico	171 (NR/NR)	0–15	36	21.00%	NR	NR	NR
Avila-Agüero et al.	2001	Costa Rica	82 (55/27)	NR	37	45.12%	NR	NR	NR
Kerrigan (Cohort B) et al.	1992	Ecuador	312 (NR/NR)	NR	38	12.17%	NR	-Gram-positive bacteria: 8/11 -Gram-negative bacteria: 3/11	-*Proteus* species: 1/11 -*Escherichia coli*: 4/11 -*Serratia* species: 3/11 -*Staphylococcus aureus*: 3/11

IQR = interquartile range; NR = not reported; SI = snakebite infection.

In the quality assessment of the studies with the NOS-CS, four studies had a high risk of bias, and the remaining 58 had a low risk of bias (Supplementary Table 3).

### Prevalence of snakebite infection.

**Figure 2.**
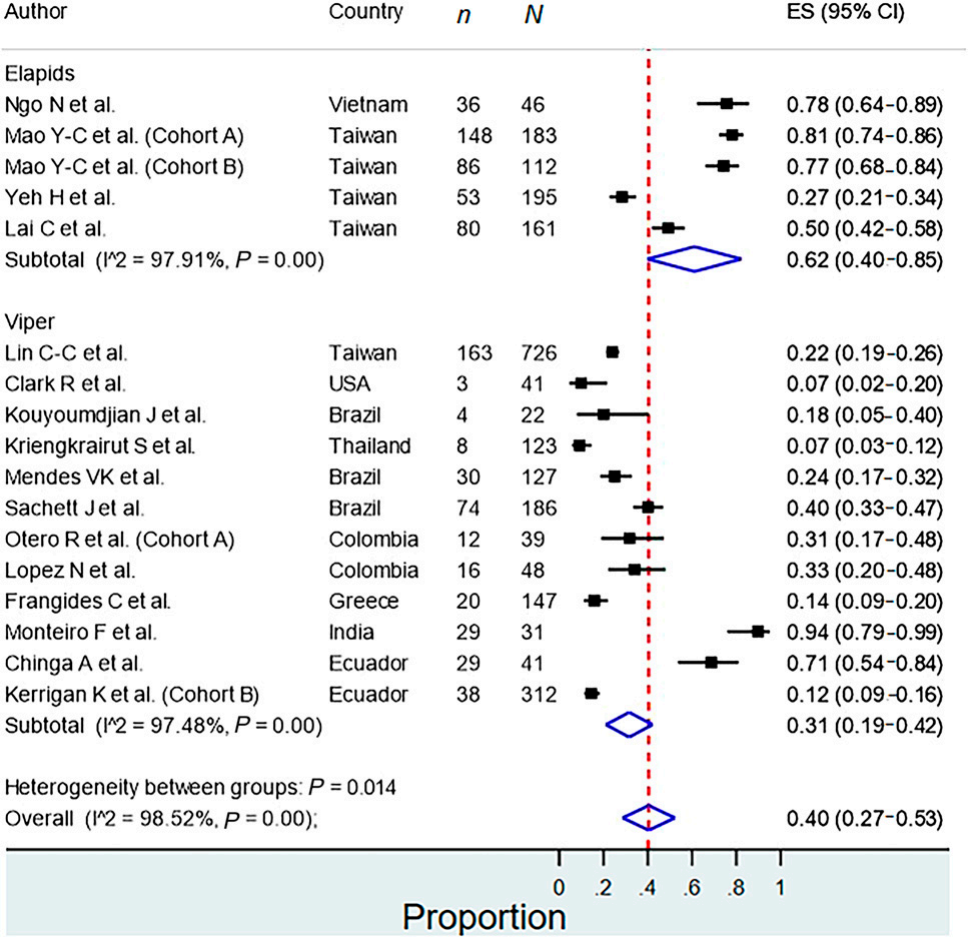
Subgroup analysis of snakebite infection according to snake families.

**Figure 3.**
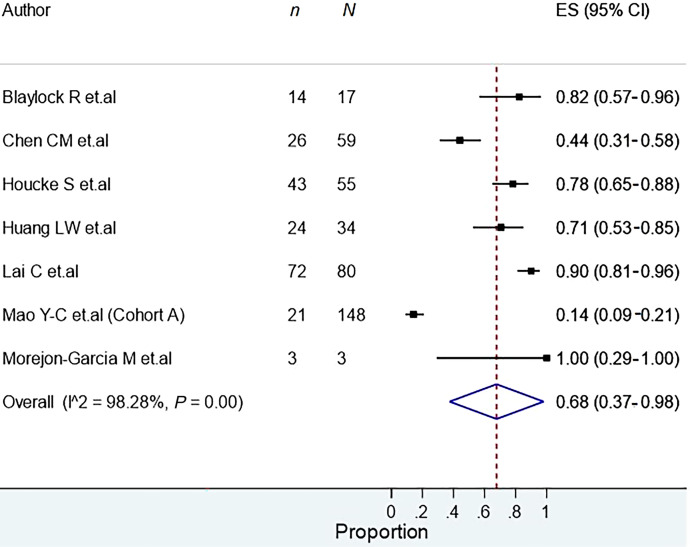
Prevalence of surgical interventions in snake bite infections.

All the meta-analyses are summarized in Table 2. The prevalence of snakebite infection was 27.0% (95% CI: 22.0–32.0%), with high heterogeneity among studies (*I*^2^ = 99.7%). In the subgroup analysis according to continents, high heterogeneity was found, and the prevalence of snakebite infection in the Asian, American, and African continents was 32%, 21%, and 29%, respectively. There was also high heterogeneity in the subgroup analysis according to snake families (Figure 2), and the prevalence of infection after a bite by Elapids and Vipers was 62% and 31%, respectively. After removing the studies with a high risk of bias, the prevalence of snakebite infection in the sensitivity analysis was 28.0% (95% CI: 23.0–33.0%); however, there was no decrease in heterogeneity (*I*^2^ = 99.72%). On the other hand, the prevalence of surgical intervention in patients with snakebite infection was 68% (95% CI: 37.0–98.0%, *I*^2^ = 98.28%) (Figure 3).

**Table 2:** Results of meta-analyses of snakebite infection

Meta-Analysis	No. of Studies	Pooled Prevalence (%)	95% CI	*I* ^2^	*P*-Value
Meta-analysis of snakebite infection					
Overall prevalence	59	27.0%	22.0–32.0%	99.7%	**<0.001**
Continents		–	–		
Asia	31	32.0%	24.0–40.0%	99.08%	**<0.001**
America	24	21.0%	13.0–29.0%	99.86%	**<0.001**
Africa	4				
Family of snakes		–	–		
Elapids	5	62.0%	40.0–85.0%	97.91%	**<0.001**
Vipers	12	31.0%	19.0–42.0%	97.48%	**<0.001**
Sensitivity analysis	55	28.0%	23.0–33.0%	99.72%	**<0.001**
Meta-analysis of surgical interventions in snakebite infection					
Prevalence	7	68.0%	37.0–98.0%	98.28%	**<0.001**
Meta-analysis of bacteria isolated in snakebite infection					
Gram-positive	16	40.0%	21.0–58.0%	96.59%	**<0.001**
Gram-negative	16	63.0%	50.0–76.0%	91.76%	**<0.001**
Anaerobes	8	4.0%	1.0–7.0%	54.19%	**<0.001**
*Morganella morganii*	15	32.0%	22.0–41.0%	83.59%	**<0.001**
*Enterococcus* spp.	11	23.0%	15.0–32.0%	98.28%	**<0.001**
*Staphylococcus aureus*	9	15.0%	6.0–23.0%	72.52%	**<0.001**
*Proteus* spp.	14	8.0%	5.0–10.0%	0.0%	**<0.001**
*Shewanella* spp.	5	7.0%	1.0–12.0%	73.06%	**<0.001**
*Escherichia coli*	10	6.0%	2.0–9.0%	40.46%	**<0.001**
*Citrobacter* spp.	4	5.0%	2.0–8.0%	0.0%	**<0.001**
*Bacteroides fragilis*	6	5.0%	2.0–8.0%	0.0%	**<0.001**
*Serratia* spp.	6	5.0%	2.0–8.0%	0.0%	**<0.001**
*Aeromonas hydrophila*	8	5.0%	2.0–8.0%	14.80%	**<0.001**
*Pseudomonas aeruginosa*	5	4.0%	2.0–7.0%	0.0%	**<0.001**
*Klebsiella pneumonia*	6	3.0%	1.0–5.0%	0.0%	**<0.001**

Bold values represent the significant value of *P* <0.05.

### Prevalence of bacteria isolated in snakebite infection.

The prevalence of Gram-positive (assessed in 16 studies), Gram-negative (evaluated in 16 studies), and anaerobic bacteria (assessed in eight studies) was 40.0% (Supplementary Figure 1), 63.0% (Supplementary Figure 2), and 4.0% (Supplementary Figure 3), respectively. Assessment of the prevalence of each isolated bacteria was: *M. morganii* (32.0%, Supplementary Figure 4), *Enterococcus* sp. (23.0%, Supplementary Figure 5), *Staphylococcus aureus* (15.0%, Supplementary Figure 6), *Proteus* sp. (8.0%, Supplementary Figure 7), *Shewanella* sp. (7.0%, Supplementary Figure 8), *E. coli* (6.0%, Supplementary Figure 9), *Citrobacter* sp. (5.0%, Supplementary Figure 10), *Bacteroides fragilis* (5.0%, Supplementary Figure 11), Serratia spp. (5.0%, Supplementary Figure 12), *Aeromonas hydrophila* (5.0%, Supplementary Figure 13), *Pseudomonas aeruginosa* (4.0%, Supplementary Figure 14), and *K. pneumonia* (3.0%, Supplementary Figure 15).

## DISCUSSION

Snakebite is considered a high-priority neglected tropical condition categorized by the WHO.^81^^,^^82^ Furthermore, snakebite has been identified as a poverty-related illness that necessitates increased awareness and collaboration worldwide to develop measures that effectively reduce the economic burden with high impact in rural tropical areas and also, to a lesser extent, in urban zones,^83^^–^^85^ as well as in travelers from multiple nontropical countries.^81^^,^^86^^–^^88^ Although most of their overall assessment has been focused on its clinical consequence, given the acute phase of envenomation, fewer studies have focused on the bite’s infectious consequences. Overall most studies focused on the clinical consequences of snakebite infection in the acute phase of envenomation, with few studies evaluating the infectious consequences of snakebite.^89^

In the current systematic review, we found a considerable prevalence of infection associated with snakebite (27%, 95% CI: 22–32%), being higher in Asia (32%) than in the Americas (21%). In the case of Africa, there is a lack of studies, limiting the analysis of this region. Nevertheless, some studies, such as that carried out in South Africa in 2017, reported a prevalence of infection by snakebite of 24.39%.^9^ Another study in Ethiopia described a higher prevalence of 62.8% in a retrospective cohort study that collected data from the medical charts of 250 patients at the University of Gondar Hospital and Metema Hospital between September 2012 and August 2020.^53^ Environmental aspects and differences in exposure may influence these results, including the growing awareness of the impact of climate change on snakebite.^5^^,^^90^^,^^91^

Snakebite infection may require surgical intervention, such as surgical debridement for extensive skin and soft-tissue necrosis,^9^ as reported in most cases (67%) in the present review. Unfortunately, the studies indicating the need for surgery lack details regarding the specific type of interventions and other related characteristics.^21^ Also, a limitation of this systematic review is that, regrettably, such secondary infections are often diagnosed due to cellulitis and abscess and, in most cases, not necessarily performing a microbiological culture to identify the causative agent, then being a purely clinical diagnosis. Thus, this review shows only those who collected secretions or biopsied the site to identify the species. Additionally, most studies did not report the antimicrobials used, the time between snakebite and the occurrence of associated infection, or the grade of envenoming of each patient. In addition, a limitation of this systematic review is that such secondary infections are often diagnosed due to the development of cellulitis and abscesses without a microbiological culture to identify the causative agent in most cases, and with the subsequent management based on a purely clinical diagnosis. Thus, this review included only studies in which secretions were collected or the bite site was biopsied to identify the bacterial species. Additionally, most studies did not report the antimicrobials administered, the time between snakebite and the occurrence of associated infection, or the grade of envenomation of each patient.

The leading group of pathogens identified corresponded to Gram-negative bacteria (63%), particularly *M. morganii* (32%), and also Gram-positive cocci (40%), especially *Enterococcus* sp. (23%) and *S. aureus* (15%). However, multiple other pathogens, also including anaerobes, were found. The pathogens were related to the type of snake mouth microbiota. For example, in some studies, *A. hydrophila* (5% in this systematic review), *M. morganii*, *K. pneumoniae* (3%), *Bacillus* sp., and *Enterococcus* sp. were isolated from the oral cavity of *Bothrops* sp.^92^ *M. morganii* is a Gram-negative bacillus usually present in the environment and the intestinal tracts of humans, mammals, and reptiles as microbiota. Despite its wide distribution, it is an uncommon cause of community-acquired infection and is most often encountered in the postoperative setting and as the cause of healthcare-associated infections.^93^^,^^94^ *M. morganii* is considered an opportunistic secondary invader that was originally thought to be the cause of summer diarrhea.^94^ However, this pathogen may also cause bacteremia, sepsis, brain abscesses, pyomyositis, meningitis, and pericarditis,^94^ among other infections,^33^^–^^43^ including the etiology of snakebite infections. Most studies did not indicate when coinfections or polymicrobial infections occurred. Although the prevalence of anaerobic infection secondary to snakebite was low, some pathogens should be taken into account, such as *Shewanella*, the most frequent anaerobic bacteria in this study (7%). For instance, a case series including 10 Asian patients bitten by cobras reported that all the patients were infected with *Shewanella*, with most presenting moderate to severe local envenomation and polymicrobial infection. However, all patients had favorable outcomes after administration of antibiotic treatment according to the antimicrobial susceptibility pattern,^95^ which is of paramount importance for the selection of adequate antimicrobial treatment.

Although we could not assess it given the lack of studies, evaluating the antimicrobial susceptibility profile of the bacterial isolates from snakebite infections would be interesting and is necessary. Because many pathogens would be associated with severe infections, it is a matter of concern, including isolating potentially antimicrobial-resistant pathogens. Although the lack of studies did not allow evaluation of the antimicrobial susceptibility profiles of the bacterial isolates from snakebite infections, it would have been of interest, especially in relation to the potential isolated of antimicrobial-resistant pathogens. Rational use of antimicrobials should be recommended. The isolation and identification of possible bacteria in snakebite wounds should be recommended in all cases to confirm or rule out an associated infection.

On the other hand, this study did not assess which snakes were more prone to cause infections, which would be helpful in clinical practice. Interestingly, a retrospective study found that cobra bites were the most frequent among patients from Taiwan.^21^ Studying which snakes are most likely to cause snakebite infections is essential for targeting therapy in low-income settings where microbiological testing is scarce.

As has been reported,^94^ the limitations of many studies include a lack of established or inconsistent criteria for an infected bite wound and the failure to use optimization techniques for pathogen isolation, especially for anaerobic organisms, which may explain the low prevalence of anaerobic infection in the present systematic review (4%). That also implies, for empirical therapy, the need to consider not only Gram-positive pathogens from human skin but also the Gram-negative and anaerobes from the snakes’ oral mouth, which may also vary according to the serpent species. For empirical therapy, this also implies the need to consider not only Gram-positive pathogens from human skin but also Gram-negative bacteria and anaerobes from the mouth of the snake, which may also vary according to the snake species. In addition, no studies from countries in Oceania were included. Nevertheless, local effects in Australian and Neo-Guinean snakebites are rare.^94^ There is also a lack of understanding of the pathogenic significance of all cultured organisms; although most of them are pathogens, their role in infection is not fully understood in all cases and clinical scenarios, and some not necessarily pathogenic bacteria may be occasionally isolated and identified. There is also a lack of understanding of the pathogenic significance of all the organisms cultured. Although most are pathogens, their role in infection is not fully understood in all cases and clinical scenarios, and some not necessarily pathogenic bacteria may occasionally be isolated and identified. Another interesting aspect would be to assess the differences in the clinical impact of snakebite infections according to the immune status of the host, including significant comorbidities, such as diabetes (e.g., *Pseudomonas* has been identified in snakebite infections), obesity, and autoimmune diseases, among others. Gathering information and conducting research more systematically and methodically through an organized research network, including zoos, veterinary practices, and rural clinics and hospitals, is needed to establish a better definition of the microbiology of animal-bite wound infections in humans, including snakebites.^94^ Because no previous systematic review has been published, the value of the current results is even higher. It is essential to highlight a clear need to develop evidence-based guidelines that include the detailed management of such associated infections.

## CONCLUSION

The prevalence of snakebite-associated infections is high, primarily due to *M. morganii*, and should be taken into account when selecting the most adequate empirical therapy. Most patients presenting with snakebite infection require surgical intervention. Rational use of antimicrobials is recommended and should guide initial empirical treatment. In light of the present results, snakebites warrant further microbiological study for the isolation and identification of bacteria in all cases to confirm or rule out an associated infection. Finally, the importance of monitoring infection in snake-bitten patients is of note.^96^

## Supplemental Materials

10.4269/ajtmh.23-0278 Supplemental Materials
