# Supplementary material for: Snakebite-Associated Infections: A Systematic Review and Meta-Analysis
Source: Am J Trop Med Hyg. 2024 Mar 19;110(5):874–86. doi: 10.4269/ajtmh.23-0278 (PMC11066359; doi:10.4269/ajtmh.23-0278)
Supplement: Supplemental Materials [file tpmd230278.SD1.pdf]

Title: Snakebite associated infections: A systematic review and meta-analysis of its prevalence

Supplementary Information

Table S1. Search Strategy

**Source**	**PubMed**
**Search**	**Formula**
**#1**	Snake Bite [MH] AND infection [MH]
**Source**	**Scopus**
**Search**	**Formula**
**#1**	TITLE-ABS-KEY (“Snake Bite” AND “infection”)
**Source**	**Web of Science**
**Search**	**Formula**
**#1**	TI=(Snake Bite AND AB=(infection))
**Source**	**Embase**
**Search**	**Formula**
**#1**	'Snake Bite'/exp AND (infection):ti
**Source**	**Scielo**
**Search**	**Formula**
**#1**	(Snake Bite) AND (Infection)

Supplementary Table S2: Full-text articles excluded

Author - Year	Title	Reason for exclusion
Abdullahi A et al. 2022	Seasonal variation, treatment outcome, and its associated factors among the snakebite patients in Somali region, Ethiopia	No prevalence of snakebite infection was reported.
Iliyasu G et al. 2015	Effect of distance and delay in access to care on outcome of snakebite in rural north-eastern Nigeria	No prevalence of snakebite infection was reported.
Thang VV et al. 2020	Incidence of snakebites in Can Tho Municipality, Mekong Delta, South Vietnam-Evaluation of the responsible snake species and treatment of snakebite envenoming	No prevalence of snakebite infection was reported.
Vongphoumy I et al. 2015	Snakebites in Two Rural Districts in Lao PDR: Community-Based Surveys Disclose High Incidence of an Invisible Public Health Problem	No prevalence of snakebite infection was reported.
Corrêa-Castro F et al. 2021	Bites by Philodryas olfersii (Lichtenstein, 1823) and Philodryas aestiva (Duméril, Bibron and Duméril, 1854) (serpentes, dipsadidae) in São Paulo, Brazil: A retrospective observational study of 155 cases	No prevalence of snakebite infection was reported.
Gendler J et al. 2021	Bites by Xenodon merremii (Wagler, 1824) and Xenodon neuwiedii (Günther, 1863) (Dipsadidae: Xenodontini) in São Paulo,	No prevalence of snakebite infection was reported.

	Brazil: a retrospective observational study of 163 cases	
De Medeiros C et al. 2019	Bites by Tomodon dorsatus (serpentes, dipsadidae): Clinical and epidemiological study of 86 cases	No prevalence of snakebite infection was reported.
Tchaou BA et al. 2016	Contribution of ultrasonography to the diagnosis of internal bleeding in snakebite envenomation	No prevalence of snakebite infection was reported.
Janes Jr D et al. 2010	Large snake size suggests increased snakebite severity in patients bitten by rattlesnakes in Southern california	No prevalence of snakebite infection was reported.
Green S et al. 2021	Epidemiology, Clinical Features, and Management of Texas Coral Snake (Micrurus tener) Envenomations Reported to the North American Snakebite Registry	No prevalence of snakebite infection was reported.
Bammigatti C et al. 2019	Serum Procalcitonin Concentration and Its Relationship with Local Manifestations after Snakebites	No prevalence of snakebite infection was reported.
Pareja R et al. 2021	Clinico-epidemiologic characteristics of ophidic accidents in a hospital of the amazonia of Peru	No prevalence of snakebite infection was reported.
Estrada-Gómez S et al. 2022	Epidemiology of Snake Bites Linked with the Antivenoms Production in Colombia 2008–2020: Produced Vials Do Not Meet the Needs	No prevalence of snakebite infection was reported.

Blessmann J et al. 2018	Incidence of snakebites in 3 different geographic regions in Thua Thien Hue province, central Vietnam: Green pit vipers and cobras cause the majority of bites	No prevalence of snakebite infection was reported.
Othong R et al. 2022	Green Pit Viper Envenomations in Bangkok: A Comparison of Follow-Up Compliance and Clinical Outcomes in Older and Younger Adults	No prevalence of snakebite infection was reported.
Chiang L-C et al. 2020	Envenomation by Trimeresurus stejnegeri stejnegeri: clinical manifestations, treatment and associated factors for wound necrosis	No prevalence of snakebite infection was reported.
Mahmood K et al. 2010	Clinical course and outcome of snake envenomation at a hospital in Karachi	No prevalence of snakebite infection was reported.
Kang S et al. 2016	Does the traditional snakebite severity score correctly classify envenomated patients?	No prevalence of snakebite infection was reported.
Michael GC et al. 2011	The effect of pre-hospital care for venomous snake bite on outcome in Nigeria	No prevalence of snakebite infection was reported.
Kularatne K et al. 2011	Revisiting saw-scaled viper (Echis carinatus) bites in the Jaffna Peninsula of Sri Lanka: distribution, epidemiology and clinical manifestations	No prevalence of snakebite infection was reported.
Karunanayake RK et al. 2014	A study of snake bite among children presenting to a paediatric ward in the main Teaching Hospital of North Central Province of Sri Lanka	No prevalence of snakebite infection was reported.

Table S3. Quality assessment of included studies

	NEWCASTLE - OTTAWA QUALITY ASSESSMENT SCALE FOR CROSS-SECTIONAL STUDIES								
	SELECTION				COMPARABILITY	OUTCOME			
STUDY	Representativeness of the sample	Sample size	Non-respondents	Ascertainment of the exposure (risk factor)	The subjects in different outcome groups are comparable, based on the study design or analysis. Confounding factors are controlled. Maximum : ☆☆	Assessment of outcome	Statistical test	SCORE	Evidence quality
Chen CM et.al	☆	☆	☆	☆	☆☆	☆	☆	*8*	Low risk of bias
Wagener M et,al	☆	☆	☆	☆	☆☆	☆	☆	*8*	Low risk of bias
Huang LW et.al	☆	☆	☆	☆	☆	☆	☆	*7*	Low risk of bias
Blaylock R et.al	☆	☆	☆	☆	☆☆	☆	☆	*8*	Low risk of bias
Ngo N et.al	☆	☆	☆	☆	☆	☆	☆	*7*	Low risk of bias

Hsieh Y-H et.al	☆	☆	☆	☆	☆☆	☆	☆	*8*	Low risk of bias
Mao Y-C et.al (Cohort A)	☆	☆	☆	☆	☆	☆	☆	*7*	Low risk of bias
Mao Y-C et.al (Cohort B)	☆	☆	☆	☆	☆☆	☆	☆	*8*	Low risk of bias
Houcke S et.al	☆	☆	☆	☆	☆	☆	☆	*7*	Low risk of bias
Lin C-C et.al	☆	☆	☆	☆	☆☆	☆	☆	*8*	Low risk of bias
Sasa M et.al	☆	☆	☆	☆	☆	☆	☆	*7*	Low risk of bias
Weed H et.al	☆	☆	☆	☆	☆☆	☆	☆	*8*	Low risk of bias
Clark R et.al	☆	☆	☆	☆	☆	☆	☆	*7*	Low risk of bias

Kouyoumdjian J et.al	☆	☆	☆		☆	☆	☆	*6*	High risk of bias
Magalhães S et.al	☆	☆	☆	☆	☆	☆	☆	*7*	Low risk of bias
Kriengkrairut S et.al	☆		☆	☆	☆	☆		*5*	High risk of bias
Osmani A et.al	☆	☆	☆	☆	☆	☆	☆	*7*	Low risk of bias
Nascimento P et.al	☆	☆	☆	☆	☆☆	☆	☆	*8*	Low risk of bias
Mendes VK et.al	☆	☆	☆	☆	☆	☆	☆	*7*	Low risk of bias
Sachett J et.al	☆	☆	☆	☆	☆☆	☆	☆	*8*	Low risk of bias
Ruha AM et.al	☆	☆	☆	☆	☆	☆	☆	*7*	Low risk of bias

Hansdak S et.al	☆	☆		☆	☆	☆	☆	*6*	High risk of bias
Villanueva Forero M et.al	☆	☆	☆	☆	☆	☆	☆	*7*	Low risk of bias
Otero R et.al(Cohort A)	☆	☆	☆	☆	☆☆	☆	☆	*8*	Low risk of bias
Otero R et.al(Cohort B)	☆	☆	☆	☆	☆	☆	☆	*7*	Low risk of bias
Lopez N et.al	☆	☆	☆	☆	☆☆	☆	☆	*8*	Low risk of bias
Yeh H et.al	☆	☆	☆	☆	☆	☆	☆	*7*	Low risk of bias
Frangides C et.al	☆	☆	☆	☆	☆☆	☆	☆	*8*	Low risk of bias
Silva A et.al	☆	☆	☆	☆	☆	☆	☆	*7*	Low risk of bias

White J et.al	☆	☆	☆	☆	☆☆	☆	☆	*8*	Low risk of bias
Yakubu A et.al	☆	☆	☆	☆	☆	☆	☆	*7*	Low risk of bias
Bhalla G et.al	☆	☆	☆	☆	☆☆	☆	☆	*8*	Low risk of bias
Looareesuwan S et.al	☆	☆	☆	☆	☆☆	☆	☆	*8*	Low risk of bias
Kumar K et.al	☆	☆	☆	☆	☆	☆	☆	*7*	Low risk of bias
Tan H et.al	☆		☆	☆	☆	☆	☆	*6*	High risk of bias
Mohammed R et.al	☆	☆	☆	☆	☆☆	☆	☆	*8*	Low risk of bias
Murugan A et.al	☆	☆	☆	☆	☆	☆	☆	*7*	Low risk of bias

Enzenhofer M et.al	☆	☆	☆	☆	☆☆	☆	☆	*8*	Low risk of bias
Ho C-H et.al	☆	☆	☆	☆	☆☆	☆	☆	*8*	Low risk of bias
Chew KS et.al	☆	☆	☆	☆	☆	☆	☆	*7*	Low risk of bias
Pradhan J et.al	☆	☆	☆	☆	☆☆	☆	☆	*8*	Low risk of bias
Kim K et.al	☆	☆	☆	☆	☆☆	☆	☆	*8*	Low risk of bias
Bhelkar S et.al	☆	☆	☆	☆	☆	☆	☆	*7*	Low risk of bias
Lai C et.al	☆	☆	☆	☆	☆☆	☆	☆	*8*	Low risk of bias
Monteiro F et.al	☆	☆	☆	☆	☆☆	☆	☆	*8*	Low risk of bias

Garg A et.al	☆	☆	☆	☆	☆☆	☆	☆	*8*	Low risk of bias
Lath V et.al	☆	☆	☆	☆	☆	☆	☆	*7*	Low risk of bias
Liu PY et.al	☆	☆	☆	☆	☆☆	☆	☆	*8*	Low risk of bias
Dookeram D et.al	☆	☆	☆	☆	☆☆	☆	☆	*8*	Low risk of bias
Kumar K et.al	☆	☆	☆	☆	☆	☆	☆	*7*	Low risk of bias
Chatterjee S et.al	☆	☆	☆	☆	☆☆	☆	☆	*8*	Low risk of bias
Ashok S et.al	☆	☆	☆	☆	☆☆	☆	☆	*8*	Low risk of bias
Miah M et.al	☆	☆	☆	☆	☆	☆	☆	*7*	Low risk of bias

Reddy M et.al	☆	☆	☆	☆	☆☆	☆	☆	*8*	Low risk of bias
Chinga A et.al	☆	☆	☆	☆	☆☆	☆	☆	*8*	Low risk of bias
Kerrigan K (Cohort A) et.al	☆	☆	☆	☆	☆	☆	☆	*7*	Low risk of bias
Saravu K et.al	☆	☆	☆	☆	☆☆	☆	☆	*8*	Low risk of bias
Morejon-Garcia M et.al	☆	☆	☆	☆	☆☆	☆	☆	*8*	Low risk of bias
Matute-Martinez C et.al	☆	☆	☆	☆	☆	☆	☆	*7*	Low risk of bias
García-Willis C et.al	☆	☆	☆	☆	☆☆	☆	☆	*8*	Low risk of bias
Avila-Agüero M et.al	☆	☆	☆	☆	☆☆	☆	☆	*8*	Low risk of bias

Kerrigan K (Cohort B) et.al	☆	☆	☆	☆	☆	☆	☆	*7*	Low risk of bias

Figure S1. Prevalence of Gram(+) bacteria

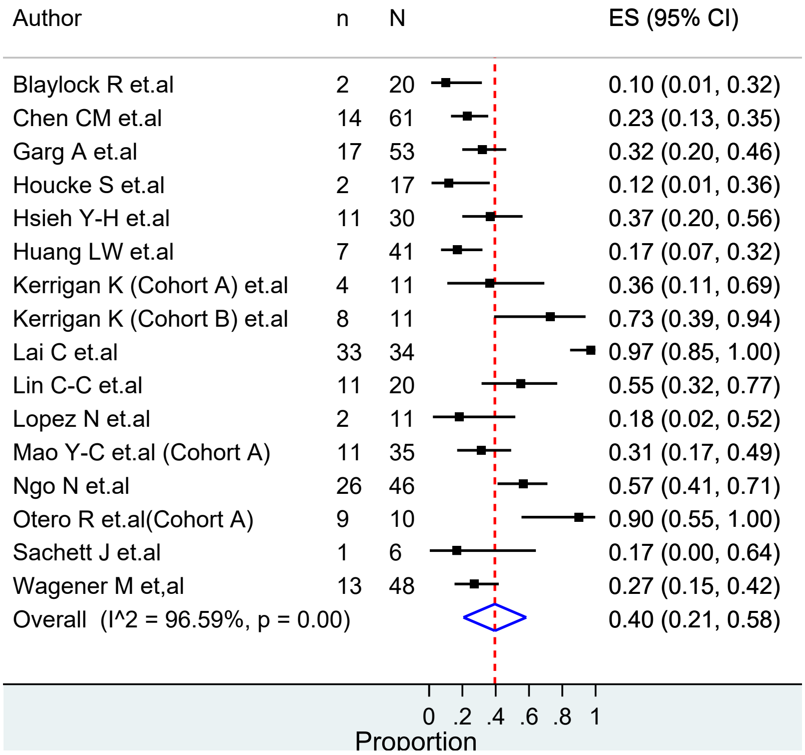

Figure S2. Prevalence of Gram(-) bacteria

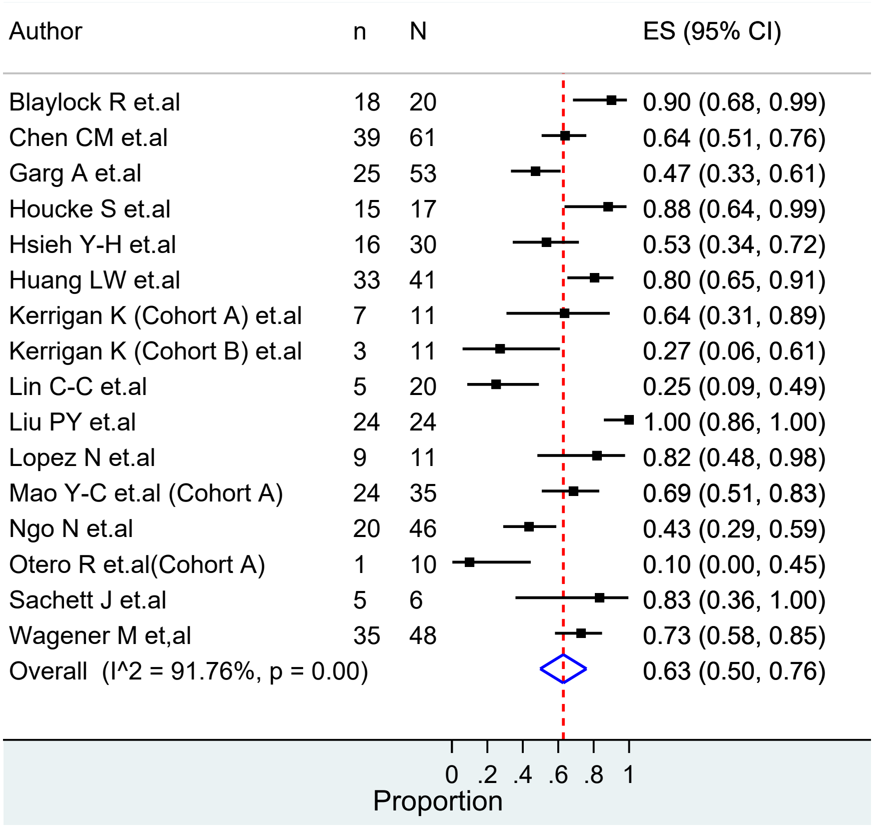

Figure S3. Prevalence of anaerobic bacteria

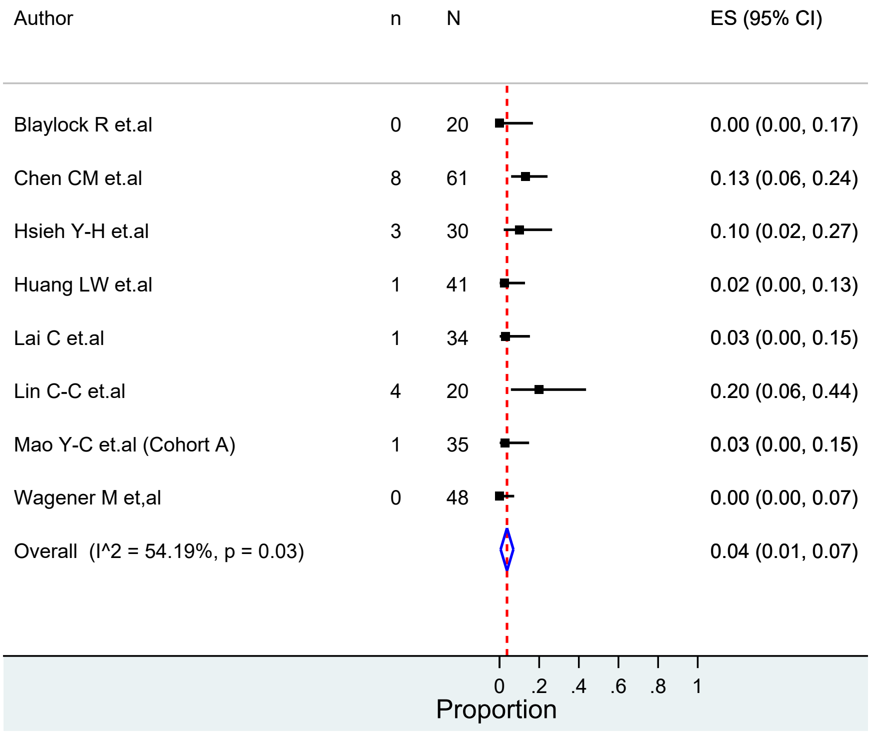

Figure S4. Prevalence of isolated Morganella morganii

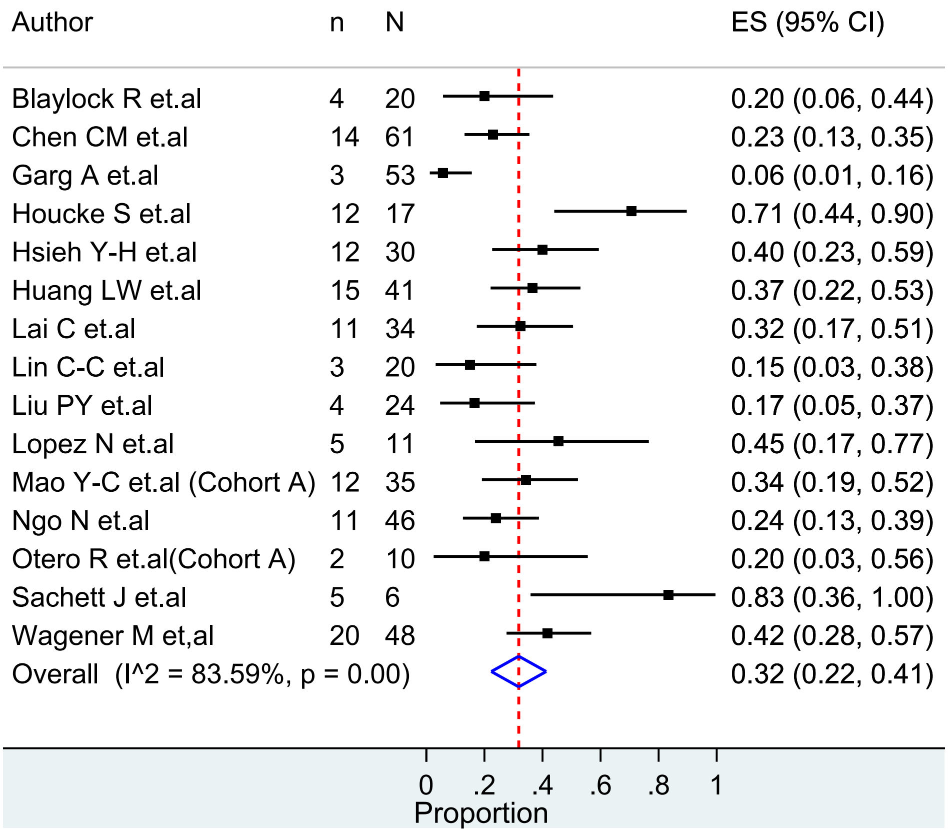

Figure S5. Prevalence of isolated Enterococcus spp

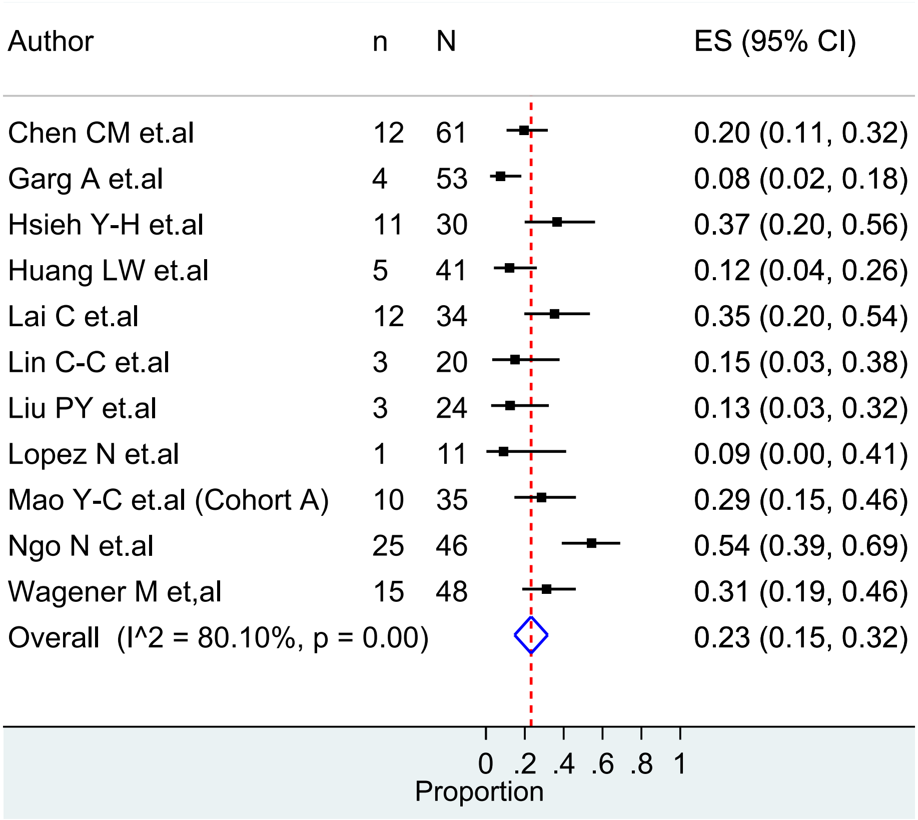

Figure S6. Prevalence of isolated Staphylococcus aureus

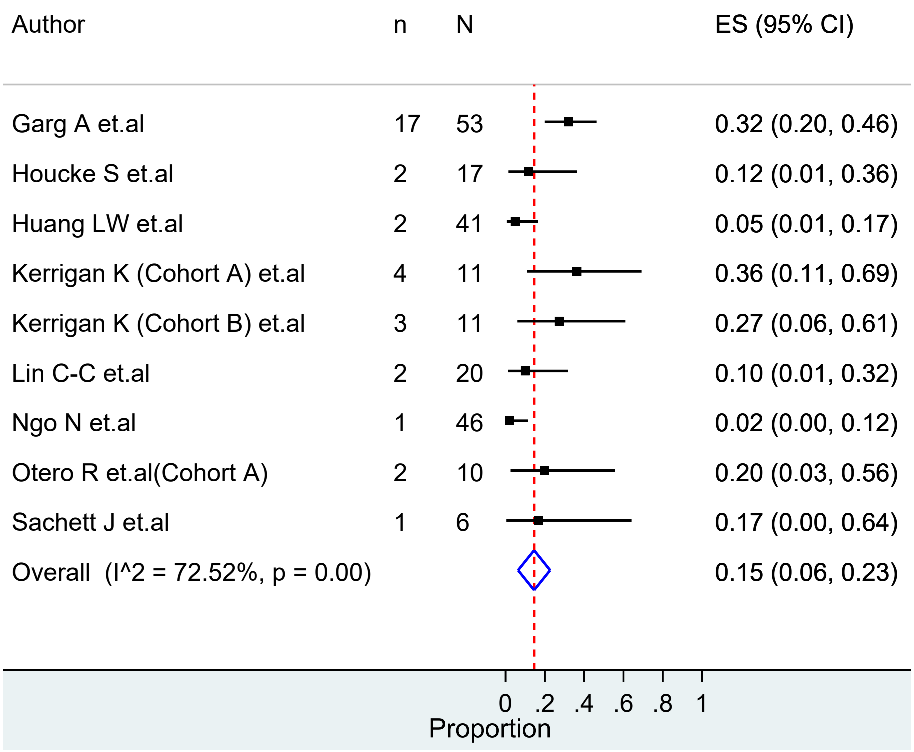

Figure S7. Prevalence of isolated Proteus Spp

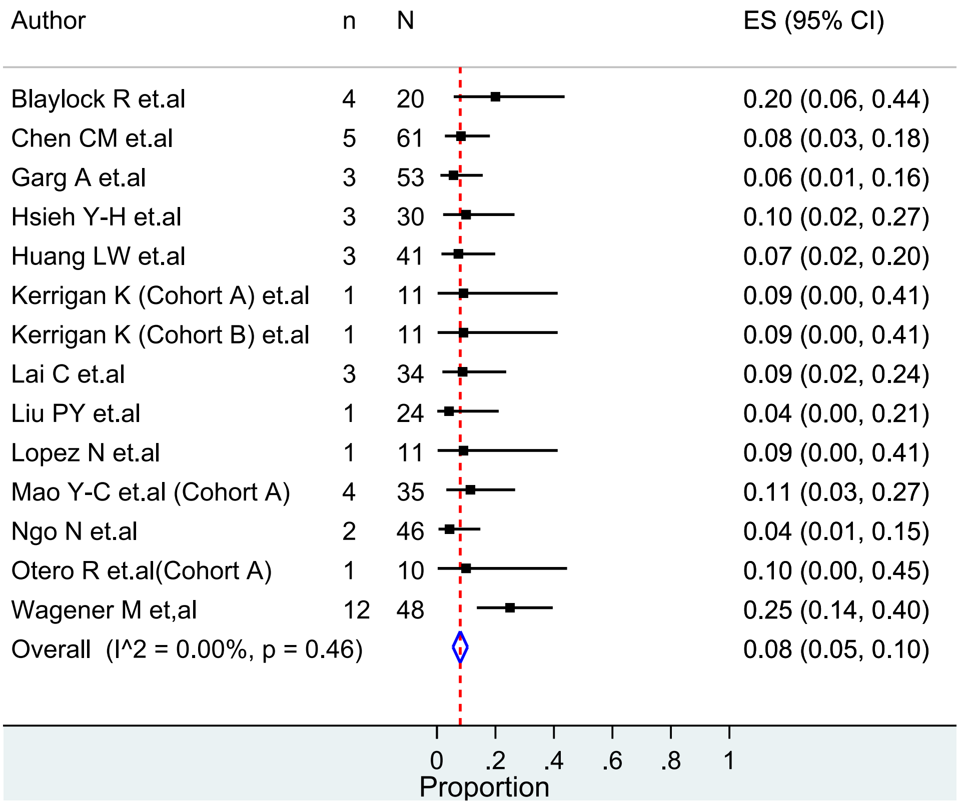

Figure S8. Prevalence of isolated Shewanella Spp

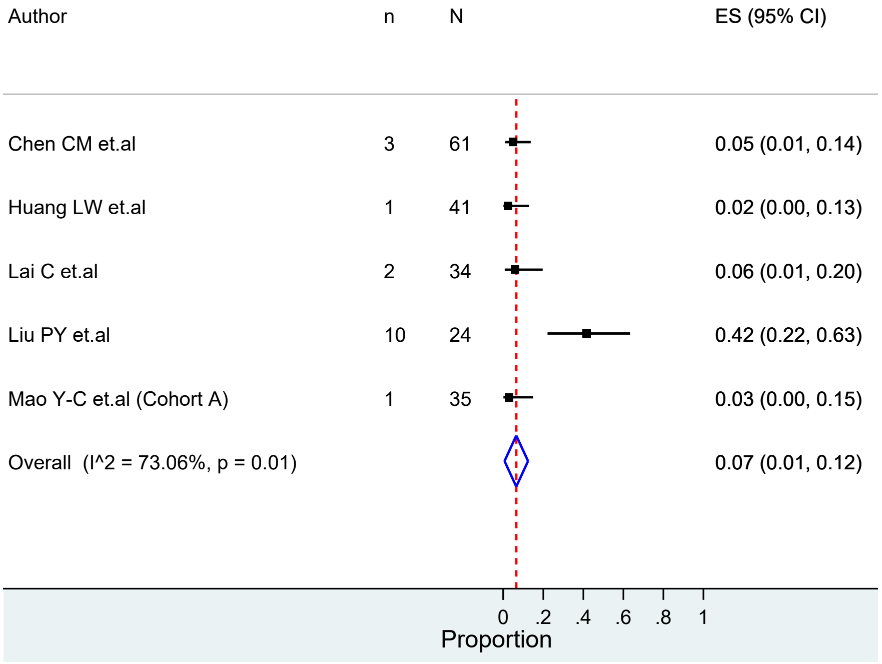

Figure S9. Prevalence of isolated Escherichia coli

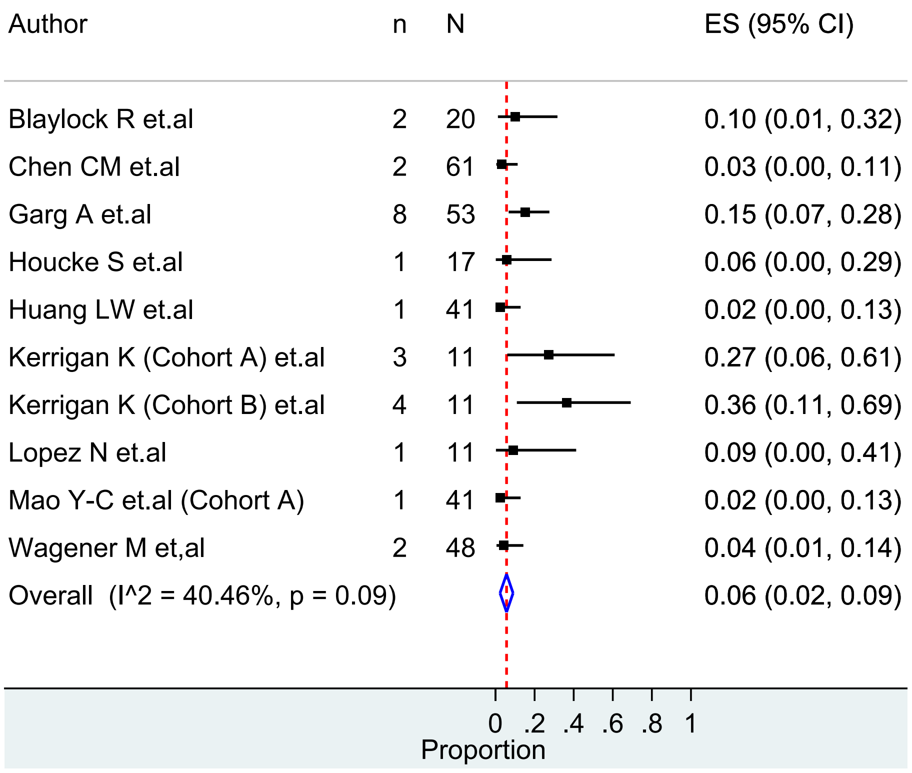

Figure S10. Prevalence of isolated Citrobacter spp

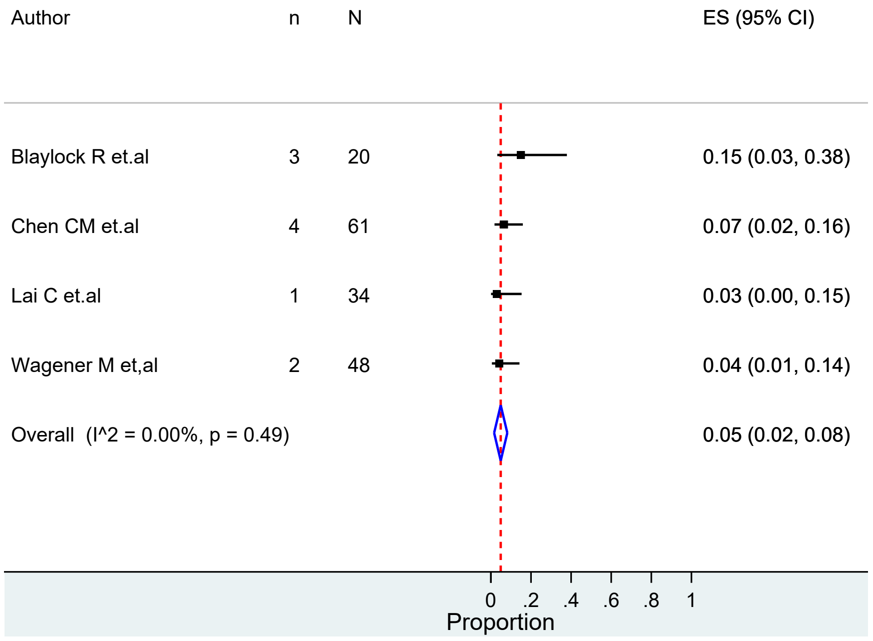

Figure S11. Prevalence of isolated Bacteroides fragilis

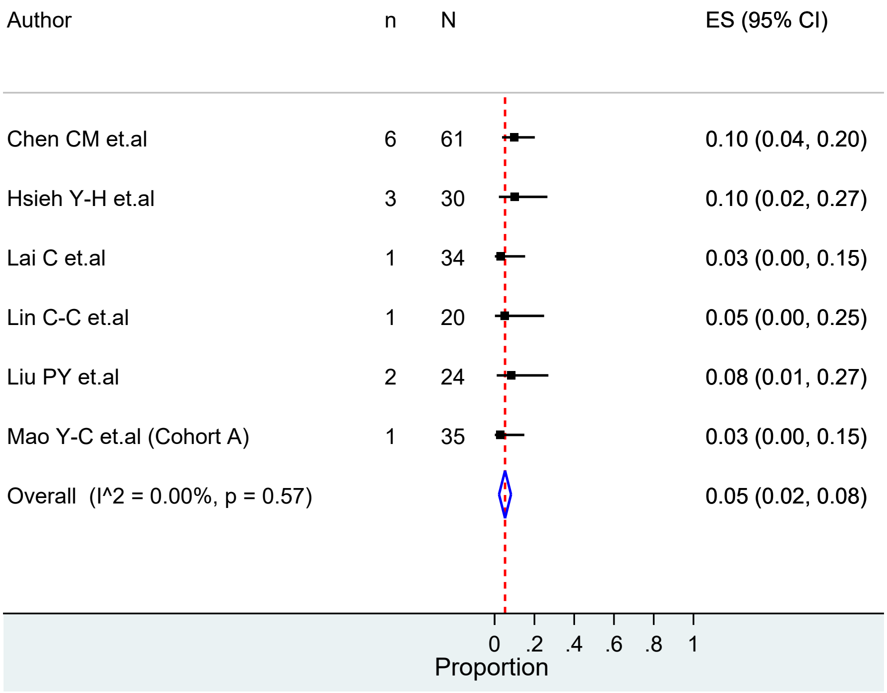

Figure S12. Prevalence of isolated Serratia spp

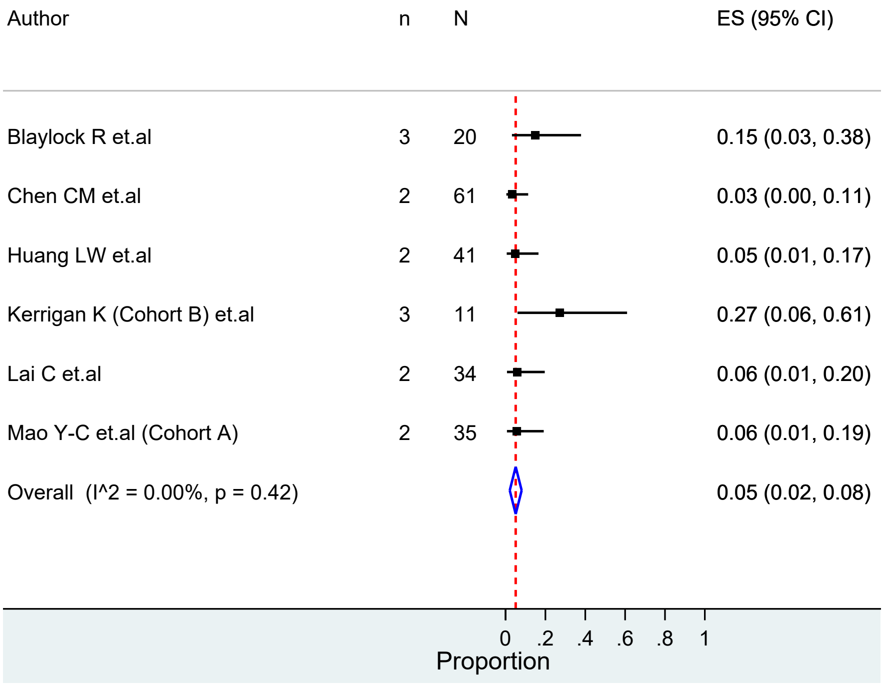

Figure S13. Prevalence of isolated Aeromonas hydrophila

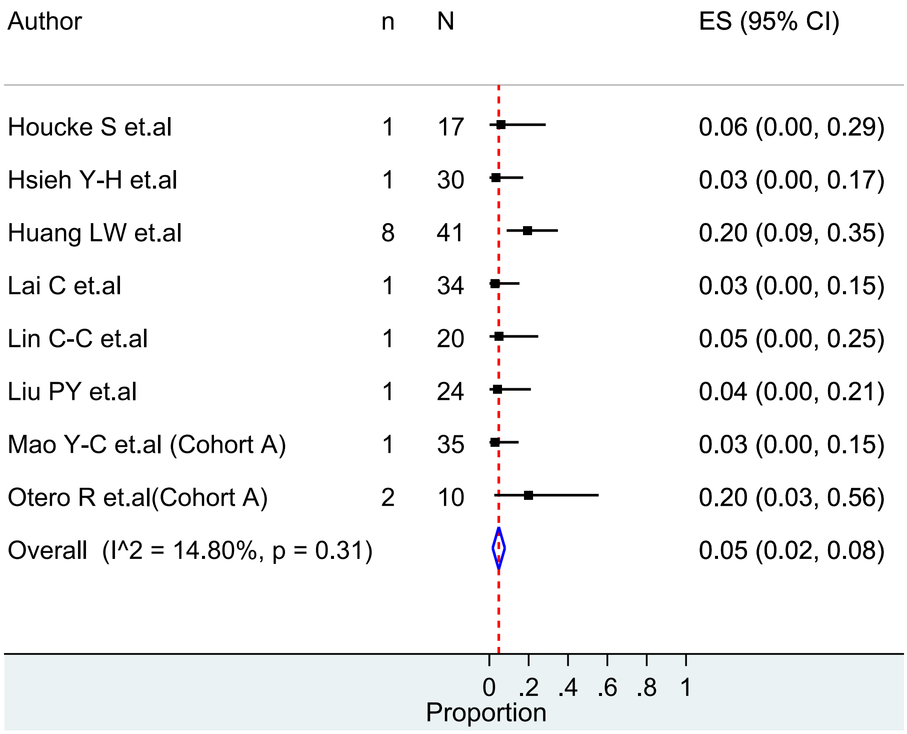

Figure S14. Prevalence of isolated Pseudomona aeruginosa

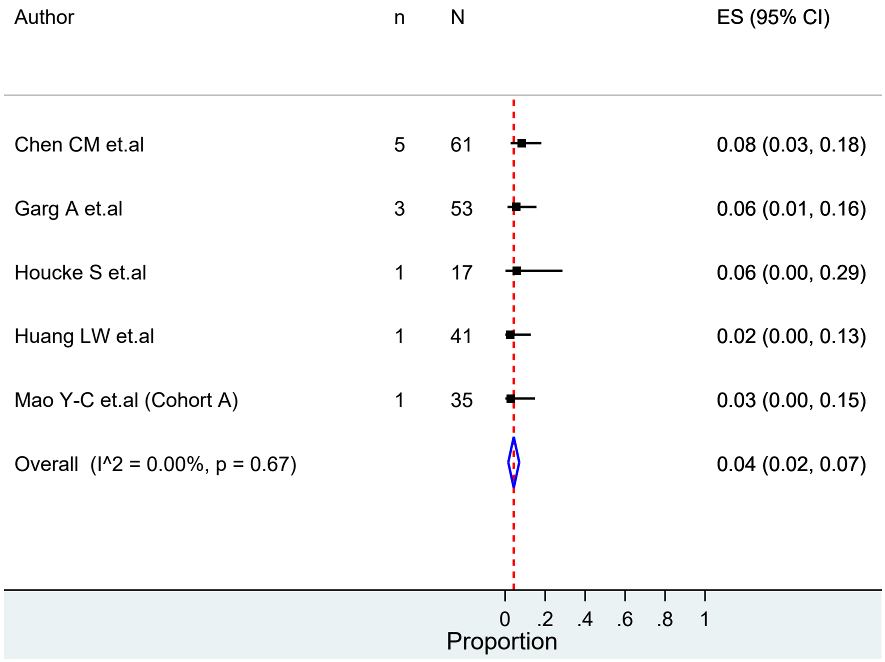

Figure S15. Prevalence of isolated Klebsiella pneumonia

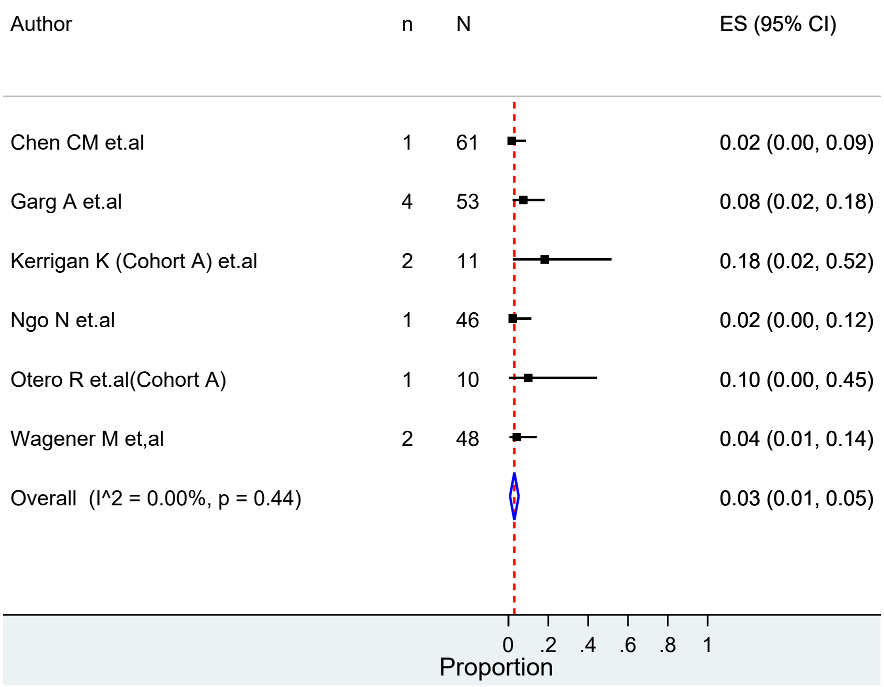
Author
n
N
ES (95% CI)
Chen CM et.al 1 61 0.02 (0.00, 0.09)
Garg A et.al 4 53 0.08 (0.02, 0.18)
Kerrigan K (Cohort A) et.al 2 11 0.18 (0.02, 0.52)
Ngo N et.al 1 46 0.02 (0.00, 0.12)
Otero R et.al(Cohort A) 1 10 0.10 (0.00, 0.45)
Wagener M et,al 2 48 0.04 (0.01, 0.14)
Overall (I^2 = 0.00%, p = 0.44) 0.03 (0.01, 0.05)
0
.2
.4
.6
.8
1
Proportion
